# Positive Indirect Antiglobulin Test in Adult Patients With Primary Warm Autoimmune Hemolytic Anemia: Clinical Significance and Prognostic Effect on Response to Corticosteroids

**DOI:** 10.1155/ah/2945671

**Published:** 2025-12-22

**Authors:** Mahmoud Husni Ayesh (Haj Yousef), Muna Al-Khalayleh, Khaled A. Abu Hmdeh, Mustafa M. Hayajneh, Weam El-Sheyab

**Affiliations:** ^1^ Department of Internal Medicine, Faculty of Medicine, King Abdullah University Hospital, Jordan University of Science and Technology, Irbid, Jordan, just.edu.jo; ^2^ Section of Blood Banking, King Abdullah University Hospital, Irbid, Jordan, kauh.jo

**Keywords:** antinuclear antibody, corticosteroids, indirect antiglobulin test, primary warm autoimmune hemolytic anemia

## Abstract

**Background:**

Primary warm autoimmune hemolytic anemia (PWAIHA) is a subtype of autoimmune hemolytic anemia (AIHA) characterized by premature destruction of red blood cells (RBCs) due to autoantibodies, typically immunoglobulin G (IgG), the cornerstone of which is a positive direct antiglobulin test. The indirect antiglobulin test (IAT) is used as a screening test to detect common and clinically significant RBC alloantibodies in patient serum.

**Objective:**

This study aimed to analyze the prevalence, clinical characteristics, and impact of positive IAT in adult patients with PWAIHA at the time of diagnosis, as well as the response to corticosteroids as the first‐line treatment.

**Methods:**

This single‐center retrospective case–control study was conducted between September 2002 and May 2024 at King Abdullah University Hospital. We analyzed data from 80 adult patients diagnosed with PWAIHA, aged a minimum of 16 years. After recording baseline investigations, including IAT and antinuclear antibody (ANA), all patients were treated with corticosteroids as first‐line treatment. The response rate and prevalence of ANA were compared between the IAT‐positive and ‐negative PWAIHA groups.

**Results:**

Baseline IAT positivity was found in 65% of our patients. Both groups were comparable in terms of age, sex, and hemoglobin levels. Serum LDH presentations were higher in positive IAT patients, response to corticosteroids was numerically higher in patients with negative IAT (54%) than those with positive IAT (33%), and after tapering steroids, patients with a positive IAT had higher rates of relapses compared with those with negative IAT. Positive ANA was found only in IAT‐positive patients (25%), which was statistically significant.

**Conclusion:**

An association between baseline IAT positivity and lower rates of complete response was observed in PWAIHA patients. The presence of IAT serves as a prognostic indicator and aids in the decision‐making process regarding treatment options. Furthermore, positive IAT results are linked to a higher prevalence of ANA positivity.

## 1. Introduction

Autoimmune hemolytic anemia (AIHA) is characterized by premature breakdown of erythrocytes caused by the production of autoantibodies [1–3]. AIHA is a rare condition, with an estimated occurrence of 1–3 cases per 100,000 individuals annually in Western countries [4]. The most common form of AIHA is warm autoimmune hemolytic anemia (WAIHA), which is primarily characterized by the interaction of immunoglobulin G (IgG) autoantibodies with red blood cells (RBCs) at a warm temperature [4].

WAIHA can be categorized into primary and secondary forms. Approximately 60% are primary, in which no underlying cause is identified, either before or at the time of diagnosis [4]. Secondary WAIHA is characterized by the presence of autoantibodies linked to other autoimmune diseases, lymphoproliferative diseases, viral infections, solid tumors, and solid organ transplantation [5–8].

Reduced hemoglobin and haptoglobin levels, elevated reticulocyte counts, unconjugated bilirubin, serum lactate, and lactate dehydrogenase levels and a positive direct antiglobulin test (DAT) indicating the presence of IgG antibodies are diagnostic laboratory indicators of primary warm autoimmune hemolytic anemia (PWAIHA) [9]. Corticosteroids are commonly prescribed as initial treatment for PWAIHA [5]. When corticosteroid treatment fails to achieve the optimum hemoglobin level or produces side effects, second‐line treatment options, such as rituximab monoclonal antibody treatment, immunosuppressive agents, and splenectomy, may be considered. Indirect antiglobulin test (IAT), alternatively referred to as the indirect Coombs test, serves as a primary method to screen for the existence of alloantibodies that may trigger RBC hemolysis. Alloantibodies are produced against antigen that is not present in the patient and can occur because of transfusion of blood products, transplantation, pregnancy, and autoimmune disease [10, 11].

The presence of alloantibodies, indicated by a positive IAT, varied between healthy blood donors and patients with PWAIHA. In healthy blood donors, the occurrence can range from 0.32% to 2.4%, whereas in patients with AIHA, it varies from 15% to 47% [12–15].

This study aimed to assess the characteristics, correlation with antinuclear antibody (ANA) positivity, outcomes, and response to corticosteroids as the first‐line treatment in patients with PWAIHA who tested positive for IAT at initial diagnosis, in comparison to those with PWAIHA who tested negative for IAT at presentation.

## 2. Material and Methods

### 2.1. Study Population

A single‐center retrospective case–control study was conducted at KAUH, a tertiary‐care national referral center for adult hematological disorders. The KAUH, with 800 beds, serves a population of approximately three million individuals in the north of Jordan [16]. This study was approved by the institutional ethical review board of KAUH. The medical records of 80 patients diagnosed with PWAIHA from September 2002 to May 2024 were recorded. Data were collected from consecutive patients diagnosed with PWAIHA. Patients were at least 16 years of age with a diagnosis of AIHA based on a hemoglobin level of less than 11 g/dL along with evidence of hemolysis. High levels of unconjugated bilirubin, high serum lactate dehydrogenase, and high reticulocyte counts were included. Bio‐Rad ID‐System Gel Card Technology was used for DAT and IAT.

In addition to the above criteria, patients were included only if they had IAT and ANA performed at the time of first PWAIHA presentation and were followed for a minimum 12‐month period. The IAT was performed as part of the standard diagnostic and clinical evaluation protocol to detect the presence of clinically significant alloantibodies prior to potential transfusion and to assess for autoantibodies that could influence disease presentation and management.

Bone marrow biopsy was conducted in patients with high suspicion of lymphoproliferative disorders and revealed only erythroid hyperplasia.

Patients who received blood transfusion or chemotherapy before diagnosis, those without IAT or ANA at the time of diagnosis or positive IAT before diagnosis, and those diagnosed with hemoglobinopathy were excluded from the study. Patients who failed to complete the 12‐month follow‐up period from the time of diagnosis, as well as those who died within this period, were excluded from the study population. Data on pregnancies prior to diagnosis were not available for the study cohort. No IgG subclass analysis was performed in this study.

### 2.2. Corticosteroid Treatment

All patients received corticosteroids as first‐line therapy. The typical initial dosage involved oral prednisolone at 1‐2 mg/kg/day, tapered gradually based on clinical response and hemoglobin levels. The tapering schedule varied by individual patient response and physician discretion but generally aimed for a reduction to a maintenance dose or complete cessation over several months.

### 2.3. Response Criteria

The criteria for complete response included achieving a hemoglobin level ≥ 12 gm/dL without the need for any further treatment within or after six months of corticosteroid treatment. This should be followed by complete tapering of medication while maintaining a stable hemoglobin (≥ 12 gm/dL), which should last for a minimum of 3 months after stopping steroids [4].

### 2.4. Statistical Analysis

All data were analyzed using SPSS for Windows Version 20 (IBM Corp., Chicago, IL, USA). Categorical data were expressed as numbers and percentages. Continuous data are expressed as median and interquartile range (IQR). All tests were two‐tailed, and a *p* value of < 0.05 was considered significant. The Mann–Whitney U test for continuous data and the *χ* ^2^ test for categorical data were used for comparisons between the two groups.

## 3. Results

**Figure 1:**
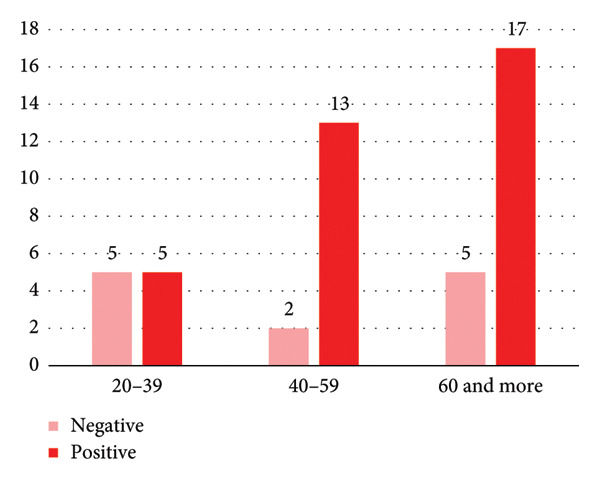
Distribution of indirect antiglobulin test (IAT) results among females by age group, showing positive and negative tests.

In this study, 80 patients were analyzed (Table 1), of whom 60% were women and 40% were men. The average age at the time of PWAIHA diagnosis was 55.75 ± 16.93 years, ranging from 23 to 92 years. The mean Hb level at diagnosis was 5.59 ± 1.29 g/dL, and reticulocyte counts were 10.9 ± 6. The median LDH level was 951 (590–1462). IAT was positive in 52(65%) (Figure 1),​ ANA ​was positive in 13(16.3%), and corticosteroid response was observed in 33 (31.3%) of the patients. No patient deaths occurred during the study period.

**Table 1:** Clinical characteristics, laboratory findings, and treatment outcomes of patients with warm autoimmune hemolytic anemia (AIHA).

Characteristics	Frequency (%)	Mean ± SD	Median (IQR)
Age (years), mean (SD)		55.75 ± 16.93	
Female/male, *n* (%)	47 (59%)/33 (41%)		
Hemoglobin (g/dL), mean (SD)		5.59 ± 1.29	
Positive indirect antiglobulin test, *n* (%)	52 (65%)		
Lactate dehydrogenase, median (IQR)			951 (590–1462)
Positive antinuclear antibody, *n* (%)	13 (16.3%)		
Steroid response (no relapse tapering steroids), *n* (%)	33 (41.3%)		
Reticulocytes (%), mean (SD)		10.9 ± 6.25	

Table 2 compares the patient characteristics based on the IAT. In both groups, patients had a mean age of 55 years. Positive IAT was detected in 65% of the patients, whereas negative IAT was detected in 35%. A positive IAT was more prevalent in females (74%). There was no significant difference when comparing the mean Hb levels, but patients with positive IAT had significantly higher serum LDH levels (*p* = 0.045). There was no statistically significant difference in the response to corticosteroids between groups, although a numerically greater number of patients who were IAT negative responded. Furthermore, all patients with negative IAT tested negative for the ANA test, while 25% of the patients tested positive for ANA (*p* = 0.004).

**Table 2:** Comparison of clinical and laboratory characteristics between patients with positive and negative indirect antiglobulin tests (IATs).

Characteristics	Negative IAT *n* 28	Positive IAT *n* 52	*p* value
Age (years), mean (SD)	55.43 ± 16.5	55.92 ± 17.31	0.902
Sex, *n* (%)			0.022
Female	12 (42.9%)	35 (69.2%)	
Male	16 (57.1%)	17 (30.8) %	
Hemoglobin (g/dL), mean (SD)	6.79 ± 1.2	6.49 ± 1.34	0.321
Lactate dehydrogenase, median (IQR)	630.5 (470.25–1988)	1043 (678–1462)	0.045
Corticosteroids: No relapse/Relapse			0.171
No relapse after tapering	15 (53.6%)	16/48 (33.3%)	
Relapse after tapering	13 (46.4%)	32/48 (66.7%)	
Antinuclear antibody result, *n* (%)			0.004
Positive	0	13 (25%)	
Negative	29 (100%)	38 (75%)	

### 3.1. Positive IAT According to Sex and Age Group

**Figure 2:**
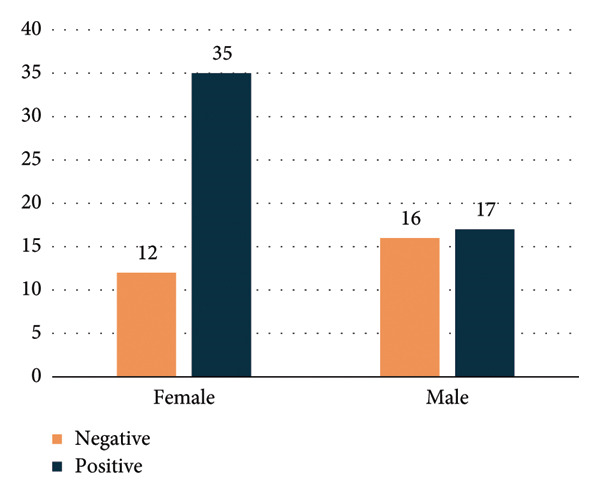
Total indirect antiglobulin test (IAT) results by sex, showing positive and negative tests.

**Figure 3:**
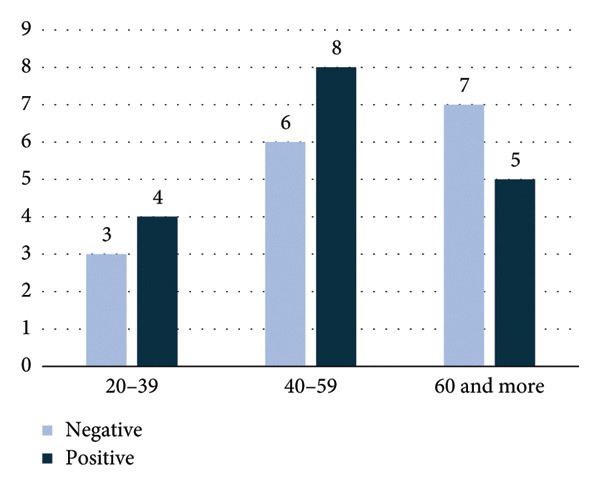
Distribution of indirect antiglobulin test (IAT) results among males by age group, showing positive and negative tests.

Regarding IAT positivity according to sex group, IAT‐positive PWAIHA in females constituted 74%, while in males (52%), as shown in Figure 2. The age distribution of females is illustrated in Figure 1, while the male age group is depicted in Figure 3.

**Figure 4:**
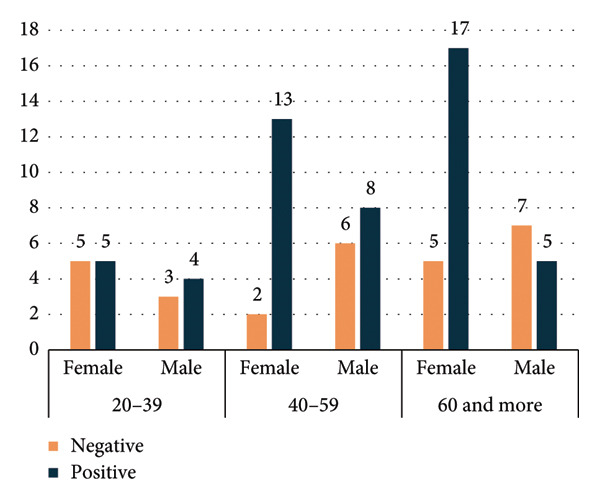
Distribution of indirect antiglobulin test (IAT) results by age intervals and sex.

Figure 4 shows the distribution of IAT results by age intervals and sex. Positive IAT results appear to cluster more prominently within specific age groups, suggesting that age may influence the likelihood of developing detectable alloantibodies or autoantibodies. Additionally, the variation between males and females indicates a possible sex‐related difference in IAT positivity.

### 3.2. Positive Indirect ANA Test According to Sex and Age Group

**Figure 5:**
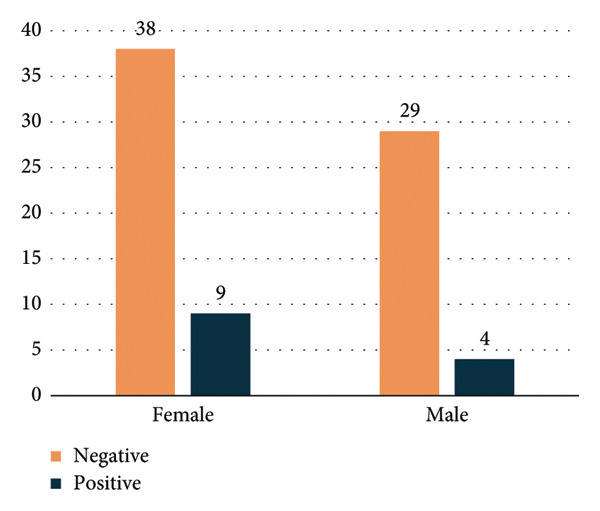
Total antinuclear antibody (ANA) test results by sex, showing positive and negative tests.

**Figure 6:**
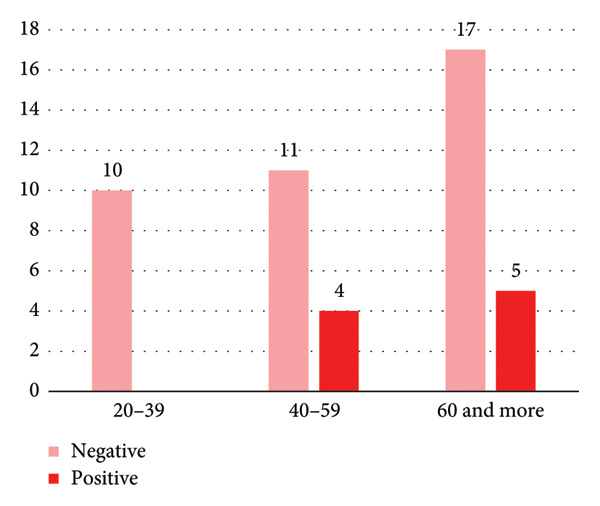
Distribution of antinuclear antibody (ANA) test results among females by age group, showing positive and negative test.

Figure 5 shows the distribution of ANA test results by sex, showing positive and negative tests and Figure 6 in females ANA positivity was more prevalent in the age group older than 40 years.

**Figure 7:**
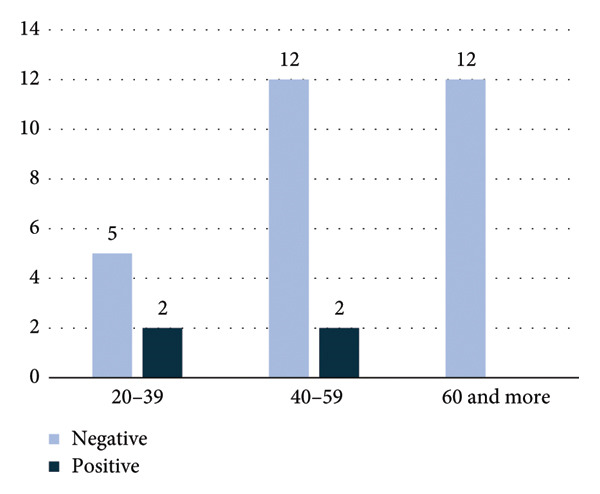
Distribution of antinuclear antibody (ANA) test results among males by age group, showing positive and negative tests.

Figure 7 Among male patients, a higher rate of ANA positivity was observed in the younger age group (< 40 years), whereas lower rates were noted in those aged 40–59 years, and no ANA‐positive cases were identified among patients older than 60 years.

**Figure 8:**
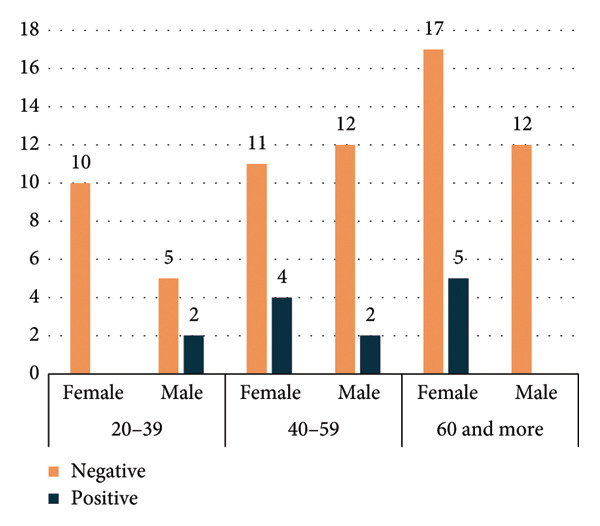
Distribution of antinuclear antibody (ANA) test results by age group and sex, showing positive and negative tests.

Figure 8 shows the distribution of ANA test results by age group and sex, showing positive and negative tests among females and male; the distribution of ANA test results demonstrates clear variation across age groups and between sexes, the differences observed between males and females suggest a possible sex‐related predisposition to ANA positivity. Overall, the figure highlights both age and sex as relevant factors influencing ANA test outcomes in the studied population.

### 3.3. Patients With Positive ANA Were Followed Longitudinally in a Separate Study

The timing of relapses was also assessed. After tapering steroids, patients with positive IAT experienced higher relapse rates than those with negative IAT. The median time to relapse was one month with a range of 1–3 months for IAT‐positive patients, compared with three months with a range of 2–4 months for IAT‐negative patients.

## 4. Discussion

This study aimed to assess the characteristics, correlation with ANA positivity, outcomes, and response to corticosteroids as first‐line treatment in patients with PWAIHA who tested positive for IAT, in comparison to those with PWAIHA who tested negative for IAT.

Our study revealed an average patient age of 56 years, which was notably higher than the age range reported in studies from India and nearly the same as that reported in Western studies [4, 17–19]. This disparity may be attributed to the exclusion of secondary AIHA, which is more prevalent among younger age group [20]. Our study findings indicated that 60% of the PWAIHA patients were female, which aligns with prior research suggesting a female predominance in this condition [4, 17, 18].

Among the individuals diagnosed with PWAIHA, 65% tested positive for IAT. A previous study showed alloimmunization between 14% and 38%, but these studies did not show the specific frequency of IAT and also did not elaborate more about the frequency according to specific subtype of AIHA, but the study did not specify the frequency in PWAIHA patients [21, 22]. IAT positivity was more prevalent in females (69%) compared with males (31%). The higher prevalence of positive IAT in females in our study may be linked to the predominance of autoimmune diseases in females; in addition, pregnancy can also stimulate alloantibody production [22–24]. As mentioned previously, patients who received any blood component transfusions prior to their PWAIHA diagnosis were excluded from our study because blood component transfusion increases the risk of alloimmunization and, hence, IAT positivity [21].

When dividing patients with positive IAT into age groups, females with positive IAT were mostly present in the age group between 40 and 59 years (86%); this could be explained by the fact that generally females in this age group generally had previous pregnancies, which increased the risk of red cell immunization [23].

A positive ANA was found in 16% of all patients and exclusively in patients with positive IAT (25%). This finding is similar to that of other studies reporting an ANA prevalence ranging from 12.6% to 58.6% in AIHA [22, 24].

Patients with positive IAT were found to have an inadequate response rate to steroids, with a higher relapse rate of 67% compared with a relapse rate of 46% in those with negative IAT. The high prevalence of ANA among patients with positive IAT may also explain the high frequency of relapses in these patients with positive IAT [24].

No statistically significant differences were observed between groups regarding hemoglobin, but serum LDH values upon presentation were higher in the positive IAT group than in the negative IAT group.

Although we focused on the IAT at the initial diagnosis of PWAIHA, it is crucial to recognize that IAT results may vary with different technical procedures [22]. Moreover, the study did not identify the quality of alloantibodies that could be responsible for positive IAT results, which may play a significant role in the response to corticosteroids as the primary treatment [25]. The retrospective nature of our study and the single‐center design are limitations, restricting the generalizability of our findings. The relatively small sample size, though typical for rare conditions, limits the power to detect subtle differences and perform extensive multivariate analyses or Kaplan–Meier survival curves, which could provide deeper insights into prognostic factors. Future larger, prospective studies are warranted to confirm these associations and explore the underlying mechanisms linking IAT positivity, ANA, and treatment outcomes in PWAIHA. The lack of IgG subclass analysis is also a limitation, as specific IgG subclasses might have different clinical implications.

In conclusion, our study indicates that baseline IAT positivity in PWAIHA patients is associated with higher LDH levels, a numerically lower response to corticosteroids, higher relapse rates, and a significant correlation with ANA positivity. These findings suggest that IAT can serve as a valuable prognostic indicator, informing clinical decision‐making and potentially identifying patients who may benefit from more intensive follow‐up or alternative therapeutic approaches.

IAT plays a crucial role in the management of patients with PWAIHA. Therefore, PWAIHA can be categorized based on positive and negative IAT results.

## Ethics Statement

This study was approved by the Institutional Review Board of King Abdullah University Hospital.

## Consent

Written informed consent was obtained from all participants prior to inclusion in the study.

## Conflicts of Interest

The authors declare no conflicts of interest.

## Author Contributions

Mahmoud Husni Ayesh (Haj Yousef) conceptualized the manuscript; performed the data analysis; wrote the original manuscript draft; contributed to writing, review, and supervision; and conducted the final critical review of the manuscript.

Muna Al‐Khalayleh collected and organized the study data.

Weam El‐Sheyab developed and performed the blood bank methodology. Khaled A. Abu Hmdeh and Mustafa M. Hayajneh designed and prepared the graphic presentation for the manuscript.

## Funding

No funding was received for conducting this study.

## Data Availability

The datasets generated and analyzed during the current study are available from the corresponding author upon reasonable request.
